# Indoor air quality improvement and purification by atmospheric pressure Non-Thermal Plasma (NTP)

**DOI:** 10.1038/s41598-021-02276-1

**Published:** 2021-11-24

**Authors:** Prince Junior Asilevi, Patrick Boakye, Sampson Oduro-Kwarteng, Bernard Fei-Baffoe, Yen Adams Sokama-Neuyam

**Affiliations:** 1 Department of Civil Engineering, Kwame Nkrumah University of Science and Technology, Kumasi, Ghana; 2 Department of Chemical Engineering, Kwame Nkrumah University of Science and Technology, Kumasi, Ghana; 3 Department of Environmental Science, Kwame Nkrumah University of Science and Technology, Kumasi, Ghana; 4 Department of Petroleum Engineering, Kwame Nkrumah University of Science and Technology, Kumasi, Ghana

**Keywords:** Environmental sciences, Chemical engineering, Civil engineering, Applied physics

## Abstract

Non-thermal plasma (NTP) is a promising technology for the improvement of indoor air quality (IAQ) by removing volatile organic compounds (VOCs) through advanced oxidation process (AOP). In this paper, authors developed a laboratory scale dielectric barrier discharge (DBD) reactor which generates atmospheric NTP to study the removal of low-concentration formaldehyde (HCHO), a typical indoor air VOC in the built environment associated with cancer and leukemia, under different processing conditions. Strong ionization NTP was generated between the DBD electrodes by a pulse power zero-voltage switching flyback transformer (ZVS-FBT), which caused ionization of air molecules leading to active species formation to convert HCHO into carbon dioxide (CO_2_) and water vapor (H_2_O). The impact of key electrical and physical processing parameters i.e. discharge power (P), initial concentration (C_in_), flow rate (F), and relative humidity (RH) which affect the formaldehyde removal efficiency (ɳ) were studied to determine optimum conditions. Results show that, the correlation coefficient (R^2^) of removal efficiency dependence on the processing parameters follow the order R^2^ (F) = 0.99 > R^2^ (RH) = 0.96, > R^2^ (C_in_) = 0.94 > R^2^ (P) = 0.93. The removal efficiency reached 99% under the optimum conditions of P = 0.6 W, C_in_ = 0.1 ppm, F = 0.2 m^3^/h, and RH = 65% with no secondary pollution. The study provided a theoretical and experimental basis for the application of DBD plasma for air purification in the built environment.

## Introduction

Indoor air quality (IAQ) is a critical factor for environmental health safety in the built environment, which often has substandard conditions due to emissions of volatile organic compounds (VOCs)^1^. Formaldehyde (HCHO) is among the common indoor air VOC pollutants directly harmful to the eyes and respiratory system, as well as the central nervous system, which could cause symptoms such as headache, dizziness, tearing, and nausea. Even worse, extensive peer review has associated the organic pollutant to various cancers and leukemia, requiring that HCHO concentration in the indoor air space be strictly regulated at 0.1 ppm according to the World Health Organization^2,3^.

Conventionally, IAQ is improved mainly by emission source control, ventilation, and air purification^4^. Emission control and ventilation in many cases are practically inaccessible owing to the ubiquitous nature of most indoor air VOCs and since buildings have become more airtight to improve air-conditioning and heating efficiency^5^. Air purification has thus become the most promising method to improve IAQ by the removal of VOC pollutants. For example, based on the observed high reactivity exhibited by some transition metallic oxides with formaldehyde, Sekine^6^ devised a multi-layer board constituted of MnO_2_ and activate carbon dust which reacted with 0.21 ppm HCHO in a flow chamber resulting to carbon dioxide as reaction byproduct and consequential reduction of HCHO to 0.04 ppm. Tian^7^ reported a 64.3% filtering of 0.99 ppm HCHO concurrently with ozone and particulate matter by an electrostatically active polyurethane—MnO_2_ membrane. However, a host of air purification techniques including catalytic combustion, adsorption, membrane separation, biodegradation, and photocatalysis have the disadvantages of low efficiency, secondary pollution, high technical requirements, high maintenance cost, short service life and small application range. What’s more, the emergence of strict regulations on VOC emission levels poses the mandate to consider precision, high efficiency, and high accuracy in the decomposition of formaldehyde. In this pursuit, reactor efficiency, mechanism, and relevant degradation reactions are key treatment parameters^8^.

Meanwhile in recent years, research on the application of atmospheric Non-thermal plasma (NTP) generated at ambient conditions for indoor air purification has heightened mainly due to the advantages of high VOC removal efficiency, high energy efficiency, and no secondary pollution. The purification occurs mainly by mixing the VOC contaminated air with a high ionization NTP containing energetic electrons and active particles which proceed to numerous inelastic collisions with the VOC molecules leading to degradation and subsequent conversion to CO_2_ and H_2_O. The NPT generation involves the oxidation and ionization of air molecules (O_2_, N_2_, and H_2_O) by high voltage induced energetic electrons to form reacting radicals such are OH, HO_2_, O, N, and H along with other active particles such as O_3_, and H_2_O_2_ energetic enough to attack organic molecules. However, some challenges associated with this emerging technology include low mineralization efficiency, generation of some undesirable byproducts such as ozone (O_3_) and NO_x_ (NO and NO_2_), and in a few NPT types low energy efficiency^9,10^.

Dielectric barrier discharge (DBD) is a good source of atmospheric NTP with low-cost plasma for indoor air VOC pollution control. DBD has the reputation to efficiently remove low concentrations of VOCs and odorous compounds plus the added advantage of energy saving and no production of dioxin^11^. Technically, the basic structure of DBD plasma reactor is constituted of a thin dielectric layer sandwiched between discharge electrode plates, forming a discharge gap and a uniformly distributed filament shaped micro-discharge pulses when operated under high voltage. Selecting a suitable dielectric material is the key to avoid the formation of sparks and to prevent corrosion of electrodes. Meanwhile, the micro-discharge thus creates an excited medium which causes the molecules of the background gas to be ionized, and due to the high oxidation strength of the active species thus formed, a consequential chain reaction is initiated leading to the degradation of different complex-shaped organic pollutants to form CO_2_ and H_2_O, making the DBD plasma system a green oxidation reactor^9–11^. In comparison with other NTP air purification systems like the corona discharge^12^, glow discharge^13^, and microwave discharge^14^ the discharge formed in the strong ionization DBD plasma system is evenly distributed, diffused, and steady. Additionally, compared with other IAQ improvement systems, the DBD plasma technology has the advantage of quick-process cycle, high operation efficiency, and no secondary pollution^11^.

Researchers have studied and reported NTP application for the improvement of IAQ. Fan^15^ developed a cylinder-shaped MnO_x_—Al_2_O_3_ catalyst aided NTP system, operated by a 25 kV DC power supply to remove 2.1 ppm formaldehyde in air. The study realized that removal efficiency increased from 42–57% when air humidity was increased from 0 to 70% RH, indicating the important role of OH radical generated from H_2_O breakdown, and the consequential reduction of ozone. However, achieving a high removal is of critical relevance for indoor safety. Zadi^16^ reported the synergistic effect of NTP with photo-catalysis process for indoor air treatment in refrigerated food chambers by removing a mixture of 0.03–0.2 ppm propionic acid and benzene as target VOCs. Even though a high removal efficiency was achieved, energy efficiency and operation cost is a major set-back. Yuan^17^ efficiently removed 0.01 ppm formaldehyde by a combined MnO_2_ catalyst-assisted NTP with physical adsorption. However, catalyst application in NTP indoor air treatment have shown poisoning effect and secondary pollution^11,18^. Additionally, Lo^19^ studied the performance of a VOC sensor-based NTP system for the removal of 0.2 ppm HCHO at different heights in a tight room, recording 80% and 73% at 1.07 m and 1.8 m respectively. The study further showed that, ozone concentration evolved from the reactor was below detection limit (0.01 ppm). This paper adapted the model of an atmospheric strong ionization DBD plasma reactor designed in a previous study by Asilevi^11^ for VOC degradation, which reported 95% removal efficiency of HCHO in synthesized flue gas.

The aim of this study therefore, was to develop and test the performance of an enhanced dielectric electric barrier discharge plasma system for indoor air purification, by the degradation of low concentration formaldehyde (HCHO). A high voltage driver was used to enhance the DBD ionization characterized by a strong electric field strength, greater average electron energy, and high electron density. The key task was to establish an optimum protocol by the effect of electrical and physical processing parameters viz., discharge energy, initial HCHO concentration, relative humidity (RH), and flow rate, and to analyze the degradation reactions. In addition, a brief operation cost analysis is presented. The study is relevant for the commercial manufacturing of DBD plasma as a promising indoor air purification technology.

## Materials and methods

### Fabrication of the DBD reactor and experimental setup

**Figure 1:**
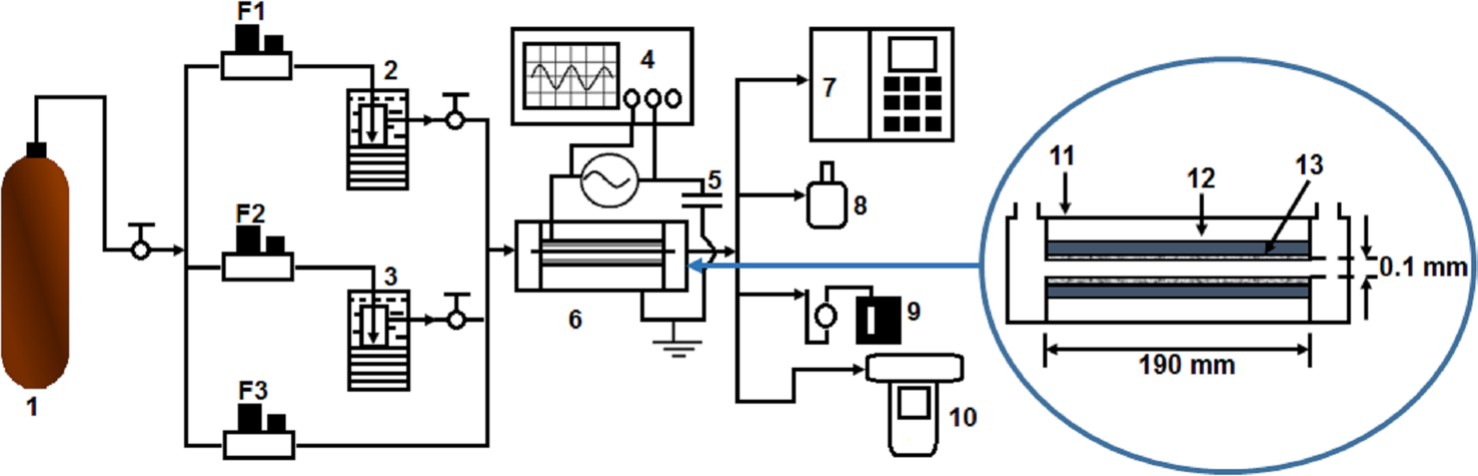
Schematic of the experimental setup. 1-dry air cylinder; 2-water-containing bubble tower, 3-HCHO-containing bubble tower, 4-oscilloscope, 5-monitor capacitor, 6-DBD reactor, 7-GC, 8-smart sensor humidity meter, 9-gas sampler, 10-aeroqual series 500, 11-PVC tube housing, 12-Steal plate electrode, and 13- aluminum oxide dielectric material. F1, F2, and F3 are the flow rate meters.

A schematic representation of the indoor air purification experimental setup to study the decomposition of low concentration HCHO is shown in Fig. 1. There are three operational components: (1) the gas flow component comprising of dry air cylinder supplying dry air through bubbler towers under flow control to regulate the air- water vapor- formaldehyde mix, (2) the reactor component comprising of the enhanced ionization dielectric barrier discharge (DBD) reactor, high voltage driver, and (3) the analysis component comprising of the smart sensor humidity meter, gas chromatography, and aeroqual series 500 HCHO detector. The experimental conditions are summarized in Table 1.

**Table 1:** Experimental measurement conditions and electrical characteristics of DBD reactor.

Parameter	Value
Reactor volume	1.9 cm^3^
Initial HCHO concentration	0.1–10 ppm
Relative humidity (RH)	20–90%
Gas pressure	≥ 0.1 MPa
Electric field intensity	10^7^ ~ 10^8^ V/m
Electron concentration	> 10^13^ m^−3^
Electron average energy	> 10 eV
Discharge power	1–5 W/m^2^

The main experimental device is the DBD reactor which generates active species by an enhanced ionization discharge system. The fabricated chamber and schematic of DBD reactor is illustrated in Fig. 1. The plasma electrodes are made from two rectangular stainless steel plates each 100 mm × 190 mm, facing each other at 0.1 mm distance using an insulating tapes, and covered by a 300 μm thick super dielectric material (SDM) made from high surface area aluminum oxide powder with dielectric constant εr ~ 10^4^. With the special characteristics of small discharge gap (l_g_) and high dielectric constant (ε_d_), a high electric field intensity (10^7^ ~ 10^8^ V/m) producing strong ionization under atmospheric pressure could be obtained according to Eq. (1)^20,21^.

1 $$ {\text{E}}_{{\text{g}}} = \frac{{{{V\varepsilon }}_{{\text{d}}} }}{{2{\text{l}}_{{\text{d}}} {\upvarepsilon }_{{\text{g}}} + {\text{l}}_{{\text{g}}} {\upvarepsilon }_{{\text{d}}}^{{\prime }} }} $$

where E_g_ (kV/cm), l_d_/l_g_ (cm), and ε are the electric field intensity, plate length, clearance, and permittivity respectively. Using a high voltage driver power supply capable of producing high voltage/high frequency of 2.5 to 7.2 kV/ 30 ~ 50 kHz between the discharge electrode and the ground electrode, the O_2_ and water vapor molecules present in the air stream are strongly ionized to form active species such as ·O, O_3_, and ·OH. Finally, the enhanced ionization DBD reactor is housed in a cylindrical PVC tube and sealed by adhesive to ensure no air leakage.

It is important to consider that, the dielectric material and its processing technology are the key to achieve a stable and enhanced electrical ionization discharge in the DBD plasma. Other researchers used dielectric materials made from glass, quartz, ceramics, polymers and other materials of relatively low dielectric and high breakdown strength^22^. This paper followed the dielectric material processing technology described by Fromille and Phillips^21^. Furthermore, in this type of DBD plasma design, almost all the electron energy (Eq. 2) acquired from the applied electric field was transferred to the air molecules.

2 $$ {\text{T}}_{{\text{e}}} = \frac{{{\sigma m}_{{\text{h}}} {\text{E}}_{{\text{g}}}^{2} }}{{3{\text{kn}}_{{\text{e}}} {\text{m}}_{{\text{e}}} {\text{v}}_{{\text{e}}}^{{\prime }} }} $$

where n_e_ (cm^−3^) represents electron concentration, and m_e_ (eV) and m_h_ (eV) are the mass of electron and heavy particles respectively, k, v_e_ (sec^−1^), σ (μS/cm), Ε_g_ (kV/cm) stand for the Boltzmann constant, electron collision frequency, plasma conductivity, and discharge intensity of the electric field, respectively.

**Figure 2:**
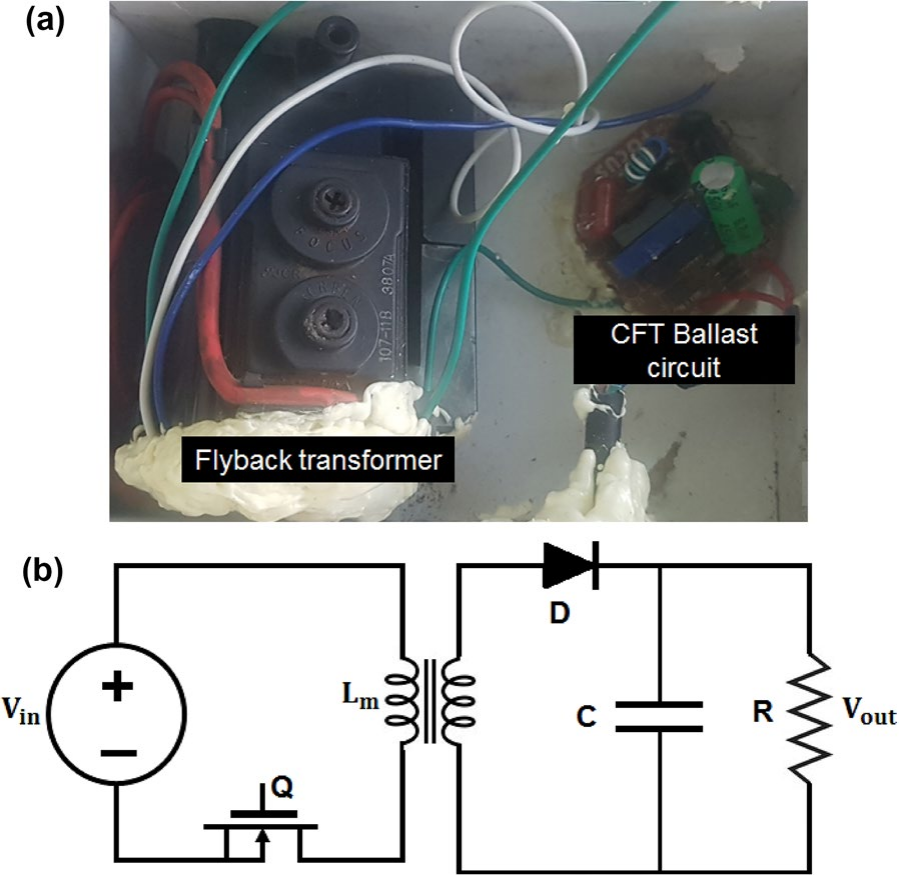
(**a**) Strong ionization voltage supply shoeing flyback transformer and CFT ballast circuit and (**b**) equivalent circuit of FBT adapted from Salem^23^.

In order to achieve a stable and strong ionization in the non-thermal plasma generated between the DBD electrodes, a zero-voltage switching (ZVS, XH-M652, 30 ~ 50 kHz) flyback transformer (FBT) with MOSFET switches and high pulsed-power output improvised from a CRT television was used (Fig. 2a). The FBT was driven by an electronic control compact fluorescent tube (CFT) ballast circuit, which is cheap and readily available, at 12 V AC input voltage (Fig. 2a) to generate a continuous electrical discharge. Figure 2b shows an equivalent circuit of the FBT; during switch on mode the diode D is reverse-biased when transistor Q conducts, and the primary winding then functions as an inductor, connected to the input source V_g_. With an input voltage of 12 V AC, the FBT is expected to generate about 7.2 kV which is 600 times the input voltage^23^.

During the non-thermal plasma discharge process, the discharge energy flow density can reach 2.1 W/cm^2^, and the average electron energy can exceed 10 eV. Thus, the system can be categorized as a strong electrically active ionization discharge, with far higher energy intensity characteristics compared to similar DBD designs, in which the dielectric barrier micro-discharge is only a local glow and the discharge intensity is weak and uniform under atmospheric pressure^24^. In this paper, the plasma discharge characteristics of the DBD reactor generates appreciably stable discharge. Moreover, the ionization intensity is comparably far higher than other atmospheric pressure discharges^25^. Table 1 summarizes the electrical characteristics of the DBD devise designed in this paper, based on the special materials used.

### Electrical measurements and calculations

As mentioned in Sect. 2.1 (Fabrication of the DBD reactor and experimental setup), the main feature of the DBD reactor relevant for the removal of HCHO is the electric discharge generated in the plasma. It is thus of practical essence to determine the discharge energy released into the system. The following computations were thus performed:

#### Degree of removal (C_out_/C_in_)

This is the ratio of final HCHO concentration after degradation to initial HCHO concentration. The degree of removal reduces as more pollutant is removed, and hence, the removal efficiency can be obtained as: 1 − (Cout/Cin)^11^.

#### Discharge power (P)

It is the average electrical power in watts (W) deposited into and consumed by the ionization reactor chamber. This is needed to calculate the SIE and to characterize the decomposition energy consumption. This paper follows the Q–V Lissajous curve method for calculating discharge power, first reported by Manley^26^, and recently shown by a host of researchers to give viable results for energy studies in DBD plasma reactor research^27^. It is given as:

3 $$ {\text{P}} = \frac{1}{{\text{T}}}\mathop \int \limits_{0}^{{\text{T}}} {\text{V}}_{{\text{r}}} \left( {\text{t}} \right) \cdot {\text{C}}_{{\text{m}}} \frac{{{\text{dV}}_{{\text{m}}} \left( {\text{t}} \right)}}{{{\text{dt}}}}{\text{dt}} = \frac{1}{{\text{T}}}\int {\text{V}}_{{\text{r}}} {\text{C}}_{{\text{m}}} {\text{dV}}_{{\text{m}}} = \frac{1}{{\text{T}}}{\oint }{\text{V}}_{{\text{r}}} dQ_{m} $$

where T is the AC cycle period, V_r_ is the high voltage across the reactor, C_m_ is the capacitance of the series capacitor also called monitor capacitor. In order to ascertain the discharge power following the Q–V Lissajous curve method, a large capacitance is be chosen relative to the reactor capacitance in order to ensure a very small voltage (V_m_) across it. Q_m_ is the charge on the capacitor. In this experiment, a 4.7 µF capacitor is connected in series with the rector as illustrated in Fig. 1. The small voltage drop (V_m_) across the series capacitor is measured with the MASTECH MY-65 digital millimetre, which is then entered into a MATLAB code to compute the charge, Q_m_. A graph of Q_m_ against V_r_ displayed on the oscilloscope is usually a parallelogram, called the Lissajous curve, whose area is the discharge energy realized in the reactor. Thus Eq. (3) can finally be written as:

4 $$ {\text{P}} = {\text{f}}{ \times }{\text{A}} $$

where f is the high voltage AC frequency in Hertz and A is the Lissajous area. The electric signals are measured by the digital oscilloscope.

### Analytical methods

Firstly, in order to study the effect of electrical discharge on HCHO degradation by the DBD plasma, a high voltage–time 500 MHz Digital Oscilloscope (WaveJet 354A) is connected across the high-voltage supply to obtain the voltage and charge waveforms. This is done by connecting the high-voltage probe (Tektronix, P6015A). The discharge current density is measured with a current probe (Tektronix, TCP303), and the root mean square (RMS) value is analyzed. The input and output concentrations of HCHO is detected by a portable air quality monitor (Aeroqual Series 500) fitted with a formaldehyde sensor head of detection range 0-10 ppm, minimum detection limit of 0.01 ppm, while the indoor air humidity (20–90%) is measured by a smart sensor humidity meter. The GCMS processes to investigate possible intermediates and degradation path is as described in Asilevi^11^.

The experiment commenced with the initial preparation of a laboratory simulated HCHO contaminated indoor air by injecting air from a dry air cylinder through water and liquid phase HCHO at controlled flow rates to generate typical contaminated indoor air with a desired HCHO concentration, which proceeds and continues to flow into the main DBD reactor for about 5 min to ensure a steady condition in the reactor ionization gas gap chamber. At a suitable steady condition, the initial HCHO concentration is noted by the portable air quality monitor (Aeroqual Series 500). The main high voltage supply system across the DBD electrodes was then turned on for plasma ionization discharge generation sustained nearly 10 min to attain a fair new HCHO concentration noted as the output concentration of HCHO. The high voltage supply is then turned off to return reactor to normal state, and the process was repeated several times for different initial concentrations. The entire process followed the description in Asilevi^11^.

## Results and discussions

### Effect of DBD strong ionization on HCHO removal

As the strength of ionization is the outstanding feature of the DBD reactor for indoor air purification, hence the removal of HCHO, this section analyzed the electrical and ionization characteristics of the DBD reactor using the digital oscilloscope and the effect on removal efficiency. The actual discharge phenomena is filamentary in nature, in which high energy fast moving electrons have collided the air molecules resulting in an electrically energy dense ionized medium comprising active species and energetic electrons, herein non-thermal plasma (NTP). Table 2 summarizes the possible reactions and active species formed in the thin plasma space. In Eqs. (5)–(8) oxygen molecules undergo ionization and dissociation in the air stream producing a large plasma volume of excited oxygen molecules and other active species. The ${\text{O}}_{2}^{ + } {\text{X}}^{2} {\uppi }_{{\text{g}}}$ and ${\text{O}}_{2}^{ + } {\text{A}}^{4} {\uppi }_{{\text{g}}}$ are unstable active byproducts proceeding to dissociation and subsequent formation of active oxygen particles such as O (^3^P), O (^2^D), and other key oxygen-based ions. Additionally, ionization (Eqs. 9 and 10) and dissociation (Eqs. 11 and 12) of nitrogen gas producing high energy electrons and consequential radicals respectively further enhances plasma strength. OH and H radical formation further results from the chemical breakdown of H_2_O represented by Eqs. (13) and (14). Atkinson^28^ and Shimizu^29^ have shown that ·OH can be formed from the collision of excited state O (^1^D) with H_2_O according to Eq. (15).

**Table 2:** Important ionization reactions involving the breakdown of air molecules to form active species in the enhanced ionization plasma.

Reaction process	K, reaction rate (cm^3^/s)	Equation numbers
e^-^ + O_2_ → ${\text{O}}_{2}^{ + } {\text{X}}^{2} {\uppi }_{{\text{g}}}$ + 2e^-^	4.2 × 10^−11^	(5)
e^-^ + O_2_ → ${\text{O}}_{2}^{ + } {\text{A}}^{4} {\uppi }_{{\text{g}}}$ + 2e^-^	9.1 × 10^−13^	(6)
e^-^ + O_2_ → O (^3^P) + O (^2^D) e^-^	4.6 × 10^−16^	(7)
e^-^ + O_2_ → O (^3^P) + O (^1^D) e^-^	3.2 × 10^−11^	(8)
e^-^ + N_2_ → ${\text{N}}_{2}^{ + } \left( {{\text{X}}^{2} {\Sigma }_{{\text{g}}}^{ + } } \right)$ + 2e^-^	1.1 × 10^−10^	(9)
e^-^ + N_2_ → ${\text{N}}_{2}^{ + } \left( {{\text{B}}^{2} {\Sigma }_{{\text{u}}}^{ + } } \right)$ + e^-^	2.7 × 10^−11^	(10)
e^-^ + N_2_ → N (^4^S) + N (^4^D) + e^-^	2.4 × 10^−17^	(11)
e^-^ + N_2_ → N (^4^S) + N (^2^D) + e^-^	2.0 × 10^−11^	(12)
e^-^ + H_2_O → e^-^ + H + OH	2.6 × 10^−12^	(13)
e^-^ + H_2_O → 2 e^-^ + ${\text{H}}_{2}^{ + }$	1.1 × 10^−12^	(14)
O(^1^D) + H_2_O → 2 OH	2.2 × 10^−10^	(15)

In the actual situation, the plasma chemical reactions and plasma formation process occurring in the strong ionization discharge reactor involve more complex reactions. Equations 5–15 mainly represent the formation of second degree electrons and key active species resulting from discharging of the indoor air. Meanwhile ·OH, O_3_, and other active species can also be produced by inelastic collision initiated by fast moving electrons with O_2_ and H_2_O molecules^30,31^. Oxygen (O_2_) gas and water (H_2_O) vapor are key deriver species in the air stream influencing the strength of NTP discharge ionization and hence controlling their concentration is relevant for device operation efficiency.

**Figure 3:**
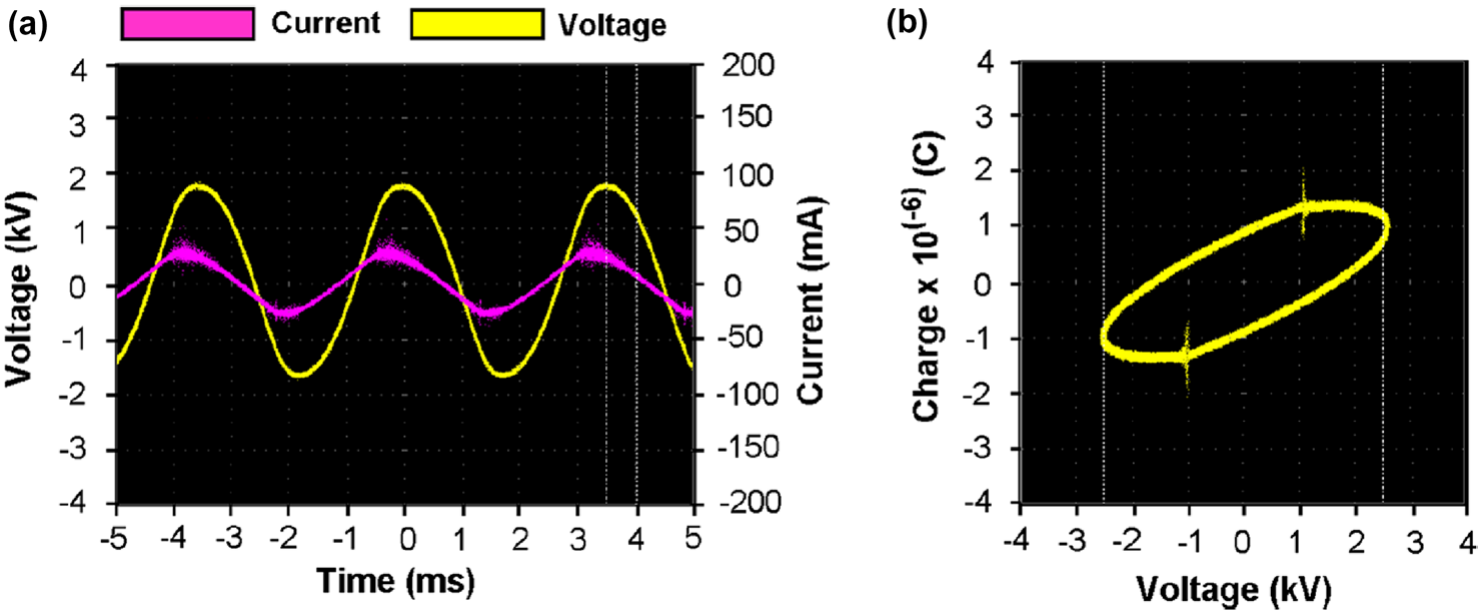
(**a**) Characteristic waveform of voltage and current in the strong ionization dielectric barrier discharge and (**b**) the charge–voltage Lissajous curve as shown on the oscilloscope screen.

Displayed on the oscilloscope screen is the characteristic waveform of voltage and current in the AC powered DBD captured in Fig. 3a, with a maximum voltage of 3.0 kV. The current waveform shows short spikes indicating micro-discharge activities in the plasma space, hence numerous discharge processes. Furthermore, the current and the voltage waveforms show a varying phase angle between them, in which the current lags behind the voltage. The charge–voltage (Q–V) Lissajous curve at 2.5 kV shown in Fig. 3b is a closed parallelogram, whose area according to Eq. (4) quantifies the discharge power.

In theory, the lumped-component electrical design of the DBD reactor in Fig. 1 resembles a lossy capacitor pair representative of the discharge electrodes, the dielectric layer, and the discharge gap, which is comparable to a resistive capacitive circuit. The design is akin to a series resonance in which the reactor terminal voltages could be several times higher than the average voltage. As supply voltage stabilizes and exceeds a specified threshold, the dielectric is electrostatically active with a suitable polarization intensity resulting in the formation a high charge density accumulation around the surface edges thus an enhanced local inhomogeneous electric field. Based on the typical voltage and current wave distributions, the electric field is computed and analyzed by ${\text{E}} = \left[ {3{\upvarepsilon }/\left( {{\upvarepsilon } + 2} \right)} \right]{\text{E}}_{{\text{o}}} \cos {\uptheta }$^11^. Here, E, ε, and θ are the localized electric field intensity, permittivity, and current–voltage phase angle.

**Figure 4:**
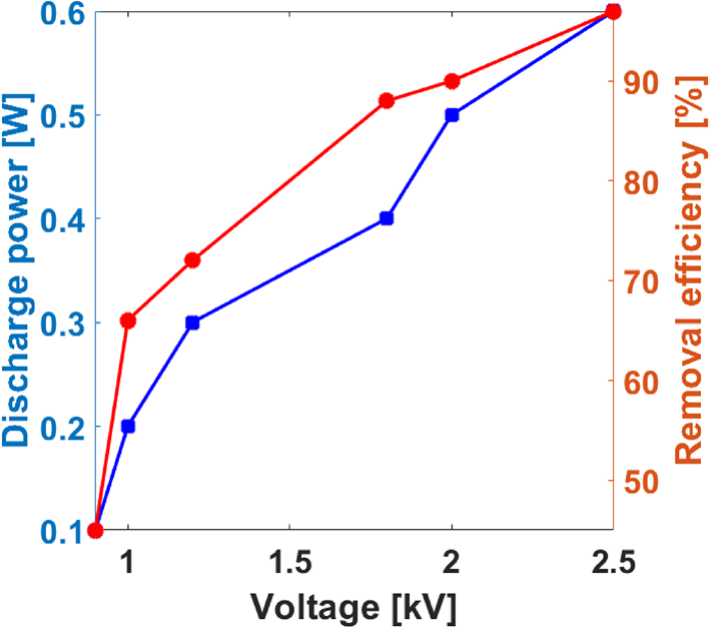
Effect of high voltage supply on ionization discharge power and removal efficiency at ambient conditions.

From this analysis, it is apparent that the electric field E relates proportionally to a threshold E_o_, such that, a larger dielectric constant will enhance E to reach three times of E_o_ when θ = 0. Thus, increasing the DBD average terminal voltage will increase the electric field intensity, which will result in large discharge power. This electrical effect is of key practical relevance in the degradation of HCHO, as an enormous quantity of high energy electrons are produced by the increased discharge power, and hence increased plasma ionization density which will increase the chance of numerous collisions with HCHO molecules thereby increasing the removal efficiency. The experimental results confirming the theory and thus agreeing with the observation of other studies^27^ is shown in Fig. 4.

In addition, Fig. 4 shows the effect of increasing discharge power by the strong ionization DBD on removal efficiency of HCHO. Formaldehyde vapor was diluted with air stream to input initial concentration of 1.0 ppm. From the experimental data, the removal efficiency of HCHO at 0.1 W was 45.4% which increased to 97% at 0.6 W under ambient conditions. Apparently, increased input voltage supplied more plasma discharge power which consequentially increased the removal efficiency of HCHO by the DBD reactor, and thus the discharge power shows positive correlation relation with removal efficiency. As mentioned, increasing the discharge power causes enhanced NTP ionization which makes available degradation active species^29^. It is worth mentioning that, in a previous study to remove HCHO by a similar strong ionization DBD reactor, Asilevi^11^ reported that increasing discharge power caused ozone generation which eventually reduced when HCHO initial concentration increased, suggesting HCHO consumption of O_3_ during the degradation process. However, removal efficiency seem to be saturated in the neighborhood of 97% with further increase in discharge power beyond 0.6 W.

### Safety of active species

The safety of active species generated by the NTP discharge has been raised in literature^32–34^. For example, Liu^33^ has reported significant correlation of negative ions generated by the widely used Negative ion air purifier with systemic oxidative stresses. Firstly, it is worth mentioning that the typical gas-phase reactive oxygen species (ROS) generated by an NTP reactor such as ·O and ·OH are short-lived by 0.1 s and 1 s respectively owing to their very constitution of unpaired electron arrangement, thus making their migration to distant space practically difficult^32,34^. However in the case of ozone (O_3_), previous experiments have shown that ozone production is significantly suppressed by VOC presence and weakly generated in ambient air alone at low supply voltage (1–3 kV) without addition of oxygen^11^. Shimizu^29^ has shown that ozone production is significantly reduced by 54% in a microplasma DBD reactor operated by a Marx generator compared with a high voltage amplifier for indoor air cleaning, while Lo^19^ reported that, using an atmospheric NTP reactor operated at 8.5 kV to decompose nearly 0.2 ppm formaldehyde, the ozone concentration evolved from the reactor was below detection limit (0.01 ppm). Additionally, the experimental results reported by Fan^15^ vis-à-vis plasma—assisted ozone evolution mechanism studies indicate that outlet ozone reduces when air humidity increases, owing to the removal of ·O by H_2_O to form ·OH (Eq. 15), which further aids formaldehyde degradation. In effect, the strong ionization type of DBD used in the present study akin to Asilevi^11^ and Shimizu^29^ has good safety.

### Effect of Initial concentration (C_in_)

**Figure 5:**
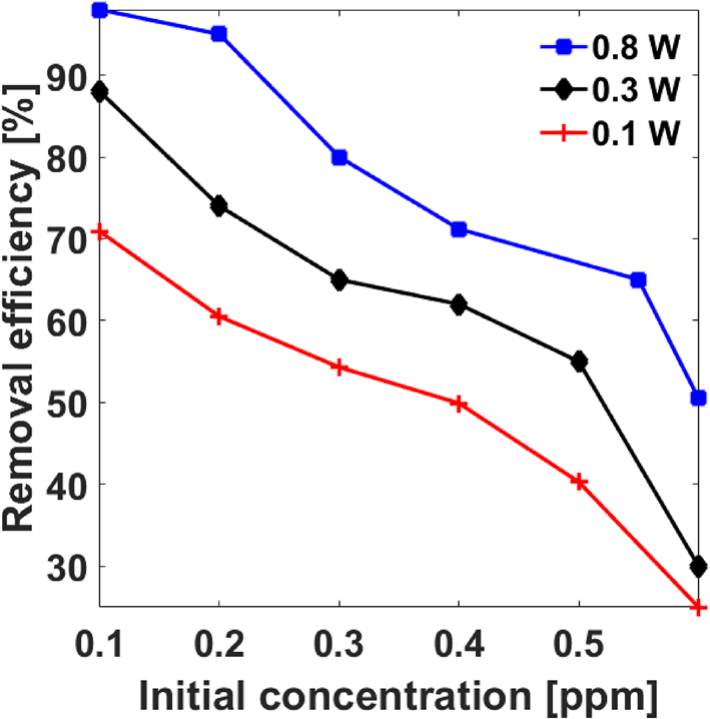
Effect of initial HCHO concentration on removal efficiency at different discharge power.

For application purpose, the effect of adjusting inlet concentration is relevant to achieve optimum removal. This section describes experimental results on the impact of initial HCHO concentration in the air stream on removal efficiency at different discharge power (0.1 W, 0.3 W, and 0.8 W) shown in Fig. 5. The inlet concentration was adjusted between 0.1 and 0.6 ppm under total air flow rate of 0.5 m^3^/h at ambient conditions. It is apparent that the removal efficiency shows a strong negative correlation with the initial concentration of formaldehyde, such that removal efficiency decreased from 70.9–25% at 0.1 W, 88–30% at 0.3 W, and 98–50.5% at 0.8 W.

Therefore, at ambient air conditions and reasonable flow rate, removal efficiency decreases with increasing initial HCHO concentration. The observation is attributed to the fact that increased inlet concentration results in increased HCHO molecule population, which reduces the chance of inelastic collisions for HCHO-electron and HCHO-active species impact owing to limited contact with individual HCHO molecules, thus tampering effective removal efficiency. Such effect was similarly reported by Hongxiang^35^ and Asilevi^11^.

### Effect of air flow rate

**Figure 6:**
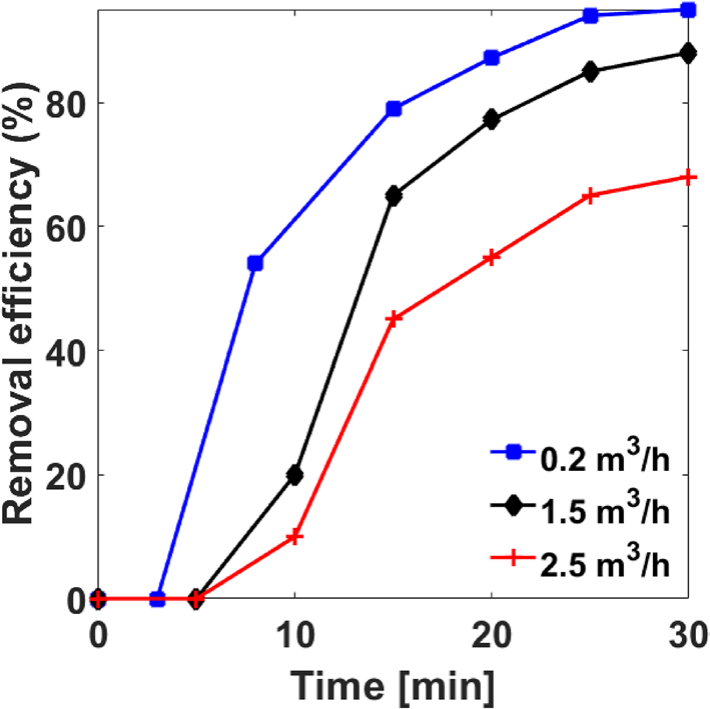
Effect of air stream flow rate on removal efficiency at different discharge power.

Since the strong ionization discharge reactor volume remains constant, the residence time expended by a specified volume of air stream in the reactor chamber is inversely related to the gas flow rate. Figure 6 shows that at any given discharge power, reducing flow rate of the air stream from 2 to 0.2 m^3^/h increases HCHO removal efficiency over increasing operation time. For example, during thirty (30) minutes operations time, the removal efficiency reached 68%, 88%, and 95% at 2 m^3^/h, 1.5 m^3^/h, and 0.2 m^3^/h flow rates respectively. This agrees with the observation of Hongxiang^35^.

In practice, as the air flow rate increases, the instantaneous reacting air volume decreases and the reaction time shortens. Although the chances of inelastic collision of gas molecules with active species and energetic electrons may increase due to high discharge power, the shortened reaction time makes more gas molecules pass directly through the reactor without any significant interaction with active species and electrons. The magnitude of the air stream residence time required to efficiently remove HCHO suggests that the prospect of tuning the reactor in order to treat large indoor air space is quite promising.

### Effect of Relative humidity (RH)

**Figure 7:**
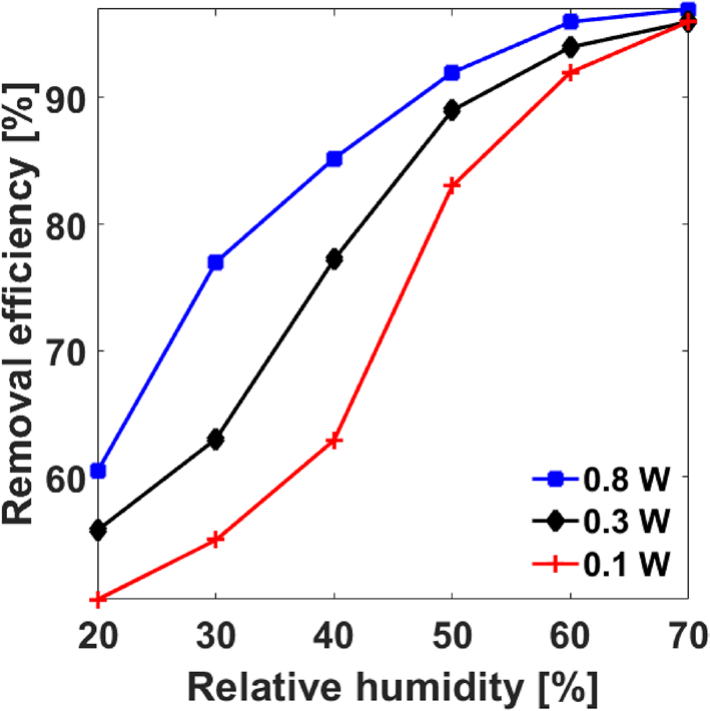
Effect of indoor air humidity on removal efficiency at different discharge power under atmospheric pressure and room temperature.

The ·OH radical which results from the breakdown of water vapor (H_2_O) due to inelastic collisions with high energy electrons and other active particles such as O (^1^D) according to Eqs. (13–15) plays a key role in bacterial decontamination and removal of VOCs in water and flue gases, because of their tendency toward strong oxidation in many physicochemical processes^36^. Studies by Shimizu^29^ and Storch^37^ reported that the destruction of HCHO by NTP in flue gas results predominantly from chemical attack by ·OH and ·O radicals. Thus this section studied the effect of water vapor on the removal efficiency of HCHO by the strong ionization DBD. Figure 7 shows that as the discharge power increased, the removal efficiency increased with increasing relative humidity (percentage of H_2_O present in air stream); for example, increasing the RH from 20 to 70% increased the removal efficiency from 50.5 to 96% at 0.1 W, 55.8–96% at 0.3 W, and 60.5–97% at 0.8 W all under atmospheric pressure and room temperature. The results show that, discharge power and RH concurrently enhanced removal efficiency of HCHO by the DBD under standard conditions.

The physiochemical effect of water vapor on the formation of plasma air stream is well documented by Shimizu^29^, Storch^37^, and Asilevi^11^. In analyzing the root mean square (RMS) current as a function of electron density in similar dielectric barrier discharge plasma system using an oscilloscope^11^, realized that increasing RH between 20 and 60% had a reducing effect on RMS current at all supply voltages, and deduced that electrons produced by the discharge process are consumed to break down H_2_O vapor molecules to generate ·OH radicals according to Eq. (13). This in turn reduces the amount of discharge electrons, thus current density. Consequentially, more ·OH radicals are formed and more HCHO molecules are destroyed increasing reactor efficiency. However, previous experiments show that, a normalization of slight deviation occurs beyond some RH limit. Chang^38^ attribute this to the fact that the electronegativity of water vapor tends to raise the minimum electric field (E∕N)_o_, where E is electric field strength and N the total air molecule number density, required to generate the plasma. According to Eq. (14), this situation multiplies electrons. This further confirms that the entire micro-discharge process is a complex one. This observation is relevant for the manufacture of DBD devises for indoor air purification in the built environment, since RH changes seasonally; an intelligent RH sensor and water vapor supply components can be fixed to regulate the air stream RH for optimum air purification.

### HCHO degradation intermediates and chemical pathway

The plasma effect on the degradation of formaldehyde was previously studied by Asilevi^11^, Shimizu^29^, and Storch^37^ and reported theoretical and experimental insights which emphasis from Gas chromatography–mass spectrometry (GCMS) and Fourier-transform infrared spectroscopy (FTIR) results that HCHO degradation starts primarily by Eqs. (16 and 17) involving ·HCO and ·OH and ·HCO and ·O inelastic collisions. In addition, direct HCHO conversion to H_2_O and O_2_ by electron collision also occurs under similar conditions. Further degradation of ·HCO occurs through collisions with high speed electrons to form relatively small molecules (including ·CH_3_, ·CH_3_CH_2_, ·CH_3_CH_2_O, ·CH_2_O, ·OHCO, ·CH_3_OH) and other organic particles. The reaction equations involved in the plasma processing degradation of HCHO are summarized in Table 3^29^.

**Table 3:** Intermediate chemical reactions in the degradation of HCHO leading to the formation of CO_2_ and H_2_O.

Reaction process	K, reaction rate (cm^3^/s)	Equation numbers
HCHO + O → HCO + OH	2.99 × 10^−11^	(16)
HCHO + OH → HCO + H_2_O	1.6 × 10^−11^	(17)
HCHO + OH → H + HCOOH	2 × 10^−13^	(18)
HCHO + H → HCO + H_2_	3.64 × 10^−16^	(19)
HCOOH + OH → H_2_O + CO_2_ + H	4.80 × 10^−13^	(20)
HCO + M → H + CO + M	8.50 × 10^−3^	(21)
HCO + H_2_ → HCHO + H	3 × 10^−18^	(22)
HCO + O_2_ → HO_2_ + CO	8.50 × 10^−11^	(23)
HCO + H → H_2_ + CO	2 × 10^−10^	(24)
HCO + O → CO_2_ + H	5 × 10^−11^	(25)
HCO + O → CO + OH	5 × 10^−11^	(26)
HCO + OH → H_2_O + CO	5 × 10^−11^	(27)
HCO + HO_2_ → OH + H + CO_2_	5 × 10^−11^	(28)
HCO + H_2_O_2_ → CH_2_O + HO_2_	1.70 × 10^−13^	(29)
HCO + H_2_O → CH_2_O + OH	3.90 × 10^−16^	(30)
HCO + HCO → CH_2_O + CO	3 × 10^−11^	(31)
HO_2_ + O → OH + O_2_	4.52 × 10^−11^	(32)
HO_2_ + OH → H_2_O + O_2_	8.00 × 10^−11^	(33)

**Figure 8:**
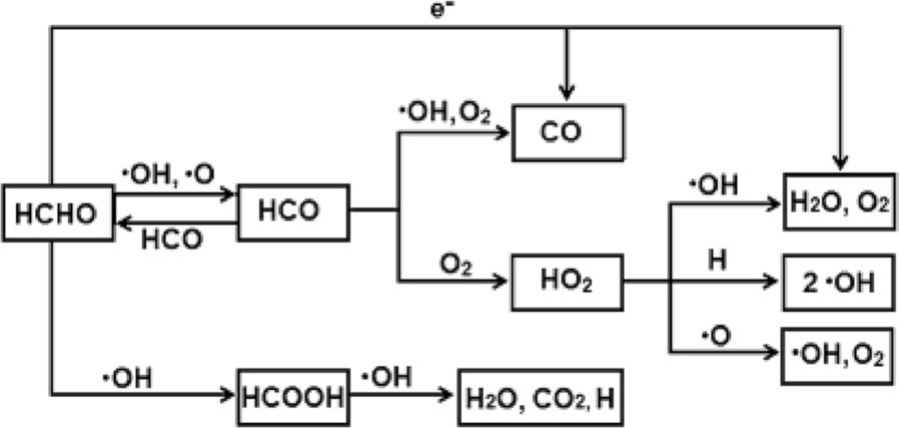
Suggested plasma processing degradation chemical path for HCHO removal in the contaminated indoor air stream.

Due to the highly active state in the plasma system during the degradation, the intermediate byproducts retain high instability. More also, the strength of oxidation of ·OH and ·O radicals generated in the strong ionization discharge plasma have strong oxidation potentials which further oxidize these intermediates byproducts. A serial plasma chemical reactions further lead to the breakdown of the small intermediate molecules and the direct electron-degraded HCHO to form CO_2_ and H_2_O. A suggested degradation chemical path is shown in Fig. 8.

### Optimization of the processing conditions

In order to ascertain an optimized condition for low-concentration HCHO removal from indoor air using the strong ionization DBD devise, several experiments under different processing conditions were performed, and sample results were selected as input data for a linear statistical regression analysis to determine which process parameters showed the strongest impact on the removal efficiency following a similar approach by Hongxiang^35^. Table 4 shows the results for the statistical regression equation analysis of the effect of different processing parameters on the removal efficiency.

**Table 4:** Statistical effect of different processing conditions the removal efficiency.

Discharge power (W)	Removal efficiency, ɳ (%)	Initial con., C_in_ (ppm)	Removal efficiency, ɳ (%)	Flow rate (m^3^/h)	Removal efficiency, ɳ (%)	Relative humidity (%)	Removal efficiency, ɳ (%)
0.1	45	0.1	85.6	2.0	28	20	55.5
0.2	66	0.2	76.5	1.5	45	30	65.0
0.3	72	0.3	66.4	0.8	64	40	75.1
0.4	88	0.4	61.0	0.6	73.1	50	88.0
0.5	90	0.55	53.4	0.4	81.3	60	94.0
0.6	97	0.6	35.2	0.2	83.7	70	96.3
y = 99.4 x + 41.53		y = -87.6 x + 94.42		y = -31.6x + 91.45		y = 0.8 x + 39.92	
R^2^ = 0.93		R^2^ = 0.94		R^2^ = 0.99		R^2^ = 0.96	

As seen, HCHO removal efficiency (ɳ) showed a strong linear correlation dependence on all the experimented processing parameters i.e. discharge power (P), initial concentration (C_in_), flow rate (F), and relative humidity (RH) of R^2^ = 0.93, 0.94, 0.99, and 0.96 respectively. However, the dependence equations with discharge power (P) and relative humidity (RH) have positive slopes of 99.4 and 0.8 respectively, while initial concentration (C_in_) and flow rate (F) have negative slopes of -87.6 and -43.7 respectively. This is expected since as discussed the removal efficiency increases with increasing discharge power and relative humidity, but decreases with increasing initial concentration and flow rate. Meanwhile, the ɳ-F regression gave the strongest correlation coefficient (R^2^ = 0.99) indicating that air flow rate in the most relevant parameter to achieve optimum degradation.

## Conclusion

In this paper, authors developed a laboratory scale dielectric barrier discharge (DBD) reactor which generates an enhanced atmospheric non-thermal plasma (NTP) to study the removal of low-concentration formaldehyde (HCHO), a typical indoor air VOC in the built environment associated with cancer and leukemia at unsafe levels (> 0.1 ppm), under different processing conditions. A strong ionization NTP was generated between DBD electrodes by a pulse power zero-voltage switching flyback transformer (ZVS-FBT), which causes ionization of air molecules leading to active species formation to convert HCHO into carbon dioxide (CO_2_) and water vapor (H_2_O). Under different processing conditions i.e. discharge power (P), initial concentration (C_in_), flow rate (F), and relative humidity (RH), the removal efficiency was determined. The results show that, (i) the DBD device efficiently removed low-concentration formaldehyde by 99% without the use of catalyst with no secondary pollution, (ii) the removal efficiency can be increased by increasing the exerted voltage hence discharge power within the range that the reactor can endure (iii) the correlation coefficient (R^2^) of removal efficiency dependence on the processing parameters follow the order F = 0.99 > RH = 0.96, > C_in_ = 0.94 > P = 0.93, iv) in the degradation process the flow rate showed the strongest impact on removal efficiency though negative, indicating a decrease in removal efficiency with increasing fume flow the efficiency reduces, and v) the processing optimization statistical results indicate that when the discharge power (P), initial concentration (C_in_), flow rate (F), and relative humidity (RH) were 0.6 W, 0.1 ppm, 0.2 m^3^/h, and 60–70% respectively, the efficiency was 99%. The experimental results are relevant in the manufacture of DBD technology for improving Indoor Air Quality (IAQ) by removing Volatile Organic Compounds.
